# Barriers to Colorectal Cancer Screening Among American Indian Men Aged 50 or Older, Kansas and Missouri, 2006–2008

**DOI:** 10.5888/pcd10.130067

**Published:** 2013-10-03

**Authors:** Aimee S. James, Melissa K. Filippi, Christina M. Pacheco, Lance Cully, David Perdue, Won S. Choi, K. Allen Greiner, Christine M. Daley

**Affiliations:** Author Affiliations: Melissa K. Filippi, Christina M. Pacheco, Lance Cully, Won S. Choi, K. Allen Greiner, Christine M. Daley, University of Kansas Medical Center, Kansas City, Kansas; David Perdue, Minnesota Gastroenterology PA, American Indian Cancer Foundation, Medicine Wheel Consulting, Minneapolis, Minnesota.

## Abstract

American Indian (AI) men have some of the highest rates of colorectal cancer (CRC) in the United States but among the lowest screening rates. Our goal was to better understand awareness and discourse about colorectal cancer in a heterogeneous group of AI men in the Midwestern United States. Focus groups were conducted with AI men (N = 29); data were analyzed using a community-participatory approach to qualitative text analysis. Several themes were identified regarding knowledge, knowledge sources, and barriers to and facilitators of screening. Men in the study felt that awareness about colorectal cancer was low, and people were interested in learning more. Education strategies need to be culturally relevant and specific.

## Objective

Our objective was to identify barriers to colorectal cancer (CRC) screening so that we could develop a culturally sensitive intervention for AI men. AI and Alaska Natives (AI/AN) are often diagnosed at later CRC stages and have higher CRC mortality rates than other US racial/ethnic groups (1,2). CRC screening is effective, but screening rates are low among AI/AN for both men and women (3,4). There may be cultural factors that would influence CRC screening and subsequent intervention strategies. For example, AI adults have reported barriers to health care that include a lack of AI clinicians and few AI elders who can serve as role models for screening (5).

### Methods

Procedures were guided by our community advisory board (CAB), made up of male and female AI/AN elders, community leaders, and community members. We conducted 5 focus groups between 2006 and 2008 with AI men (N = 29) aged 50 or older in communities and tribal areas in Kansas and Missouri. Participants were recruited through flyers, word-of-mouth, and information tables at powwows and other cultural events. The study was approved by the University of Kansas Medical Center’s Human Subjects Committee. If we recruited or collected data on tribal property, we also sought approval through the tribal council or equivalent authorities. After the study, we shared our findings with our CAB and through “community research days” open to anyone.

The moderator’s guide was based on prior work with community leaders and health providers; questions addressed knowledge and attitudes. Focus groups were held at community locations. Before the group, participants provided written informed consent and completed a brief demographic survey. A group meal was provided before the group and each participant received a gift card for $25. Group sessions lasted about an hour and were recorded and transcribed verbatim. We stopped conducting groups after saturation was achieved on major themes.

The analysis was conducted by a joint team of researchers and community members, following a community-based participatory research method developed by the team that trains community members in qualitative analysis and includes them as critical members of the project (6). We developed code books by forming an inductive list of topics and discussing topic definitions and codes. Once the code book was finalized, 3 members of the joint team used the codebook to code transcripts by hand. Approximately 10% of the codes were cross-checked to ensure intercoder reliability; few differences were found. Coders identified preliminary themes that were then combined into thematic statements by the principal investigator (C.M.D.) and checked by a community member (L.C.). All exemplar quotes were selected by community members.

## Results

Participants (N = 29) were AI men aged 50 or older (Table 1). Themes (Table 2) are described below.

**Table 1:** Demographic Information of American Indian Elder Men (N = 29) Participating in Focus Groups on Colorectal Cancer Screening, Kansas and Missouri, 2006–2008

Variable	% (n)
**Current living situation**	
Married/partnered	75.9 (22)
**Education**	
Some high school	6.9 (2)
High school graduate/General Educational Development (GED) certificate	10.3 (3)
Post-high school certification	13.8 (4)
Some college or college degree	69.0 (20)
**Health insurance outside of Indian Health Service (IHS)**	
Yes	93.1 (27)
**Where do you receive the majority of your health care?^a^**	
IHS	39.3 (11)
**Have you ever talked with your doctor about colon cancer testing?^a^**	
Yes	75.0 (21)
**Have you or any of your blood relatives ever been diagnosed with colon polyps by a doctor or nurse?^a^**	
Yes	14.3 (3)
**Have you or any of your blood relatives ever been diagnosed with colorectal cancer?^a^**	
Yes	10.7 (3)
**Have you ever had a fecal occult blood test (FOBT)?**	
Yes	31.0 (9)
**Have you ever had sigmoidoscopy?**	
Yes	24.1 (7)
**Have you ever had colonoscopy?**	
Yes	33.3 (9)

a Not all participants responded.

**Table 2:** Themes That Emerged From Focus Groups With American Indian Elder Men (N = 29) About Colorectal Cancer Screening, Kansas and Missouri, 2006–2008

Theme
**Men had general knowledge about colorectal cancer (CRC).**
*I think it would all go down to knowledge and awareness. ’Cause if you knew what could happen by not getting checked, then you’d be more motivated to get checked.*
**Knowledge came from several sources.**
*Well I don’t think it was educational stuff. It may have been just that you hear all the stories, you know that something has to happen when you’re 50.*
*I don’t know if it was advertising or reading stories about who had died of cancer. And every time that you’d read a story about it there’d be something saying that if they caught it earlier.*
**American Indian (AI) men felt that there were numerous barriers to screening.**
Cost/insurance:* Yeah, cost and no insurance would be the main thing I could see. From listening to some of the others, it sounds like access. It sounds like there’s nothing in this area for someone that don’t have insurance or anything to get it done.*
Privacy/embarrassment: *It would be embarrassing to have to go pull their pants down and let somebody do something like that to them. That in itself and their mentality… and believe me I’ve talked to them about it, they wouldn’t do that. . . . I mean I definitely think there’s an embarrassment factor there.*
Fear: *But a lot of them don’t know the procedure, don’t know nothing about it and they’re afraid to ask. So you have a lot of natives dying off because they’re afraid to go up there and ask somebody a question and we’ve got to figure out how to get this out to the public.*
**There are negative perceptions of clinicians or systems that can affect willingness to get screened.**
Vague information: *They [providers] mentioned it but then, you know, they said, well, we’d like for you to have one [colonoscopy] sometime in the near future, but that’s, you know, the extent of what. . . . They never actually told me what it is, what it involves.*
Dissatisfaction: *If somebody’s dealt with a health center, Indian Health Service, and was not happy with whatever they were doing, then that would . . . make them less likely to be proactive*.
**The majority of participants had discussed CRC only with their health care providers.**
Inadequate detail from providers: *When you do hear [a doctor’s recommendation for screening] it’s just a slight mention. You know, like when I got my physical he says, you’re over 50, I would recommend a colonoscopy, you know. But why?*
Reasons for talking about CRC: *Yeah, that’s what killed my father-in-law. You know he got sick and didn’t go to the doctor, and when he got to the point where he went to the doctor, which was only 3 months too late. He lasted about a month.*
**Traditional perspectives must be incorporated into intervention messages about CRC.**
*There are hospitals, Indian Health Hospitals on reservations that are doing that, working with the traditional medicine... And it works good when they [traditional and Western medicine] get together. You know, you have both and the Western medicine doctors respect that and, you know, they work hand in hand.*
*I still believe in traditional ways and I don’t turn down any traditional medicine or any healing ceremonies or anything like that, but I know that, you know, I can’t go to [a traditional healer] and get a colonoscopy.*
**Participants stressed that education and prevention strategies for CRC should use multiple formats. **
A suggested primary message was “*you are at higher risk [for CRC] and these are the sources that are available to you.*”

**Most men had general CRC knowledge.** Others knew risk factors, symptoms, or screening methods. Those with more knowledge often had personal experience through a friend or family member. The less information people have, the less likely they are to get screened.

**Knowledge came from television and from community events, print materials, and family.** Television was a common source. Social interaction, such as sharing personal stories, was also important. Little of the available information was specific to AI men; generic materials often overlooked cultural factors.

**Barriers to screening varied.** Insurance and cost were discussed at length. Many Indian Health Service (IHS) clinics were far away, wait-times were long, and colonoscopy was often contracted out. Participants said AI men tend to be private about certain issues; embarrassment and fear were mentioned.

**Negative perceptions of clinicians/systems affected willingness to get screened.** Some perceptions stemmed from previous experience, distrust of places where AI people receive care, or the sense that CRC information seemed vague. Navigating health care sometimes evoked dissatisfaction and disappointment, which might inhibit care-seeking.

**CRC was mostly discussed with doctors.** However, insufficient detail was a common complaint. Those who discussed it with others often had been affected by CRC or spoke to someone who was affected. Mostly, men characterized CRC discussions as uncomfortable and uncommon.

**Traditional perspectives must be acknowledged.** Traditional medicine may be used alone or alongside Western medicine. Participants indicated there might be less resistance if programs addressed cultural structures and traditional ways.

**Participants stressed that interventions should use multiple strategies and channels, but there was no consensus about which was best.** Efforts should be culturally targeted and message-bearers should be AI. Some mentioned highlighting the role of women, or tapping networks outside the family, such as at work or events such as powwows.

## Discussion

Our purpose in this study was to expand our knowledge about CRC screening among AI men. We found that AI men exhibited many commonalities with other groups. Yet our data also indicate barriers that may be specific to AI men.

Privacy and embarrassment seemed especially relevant for our participants based on how much they talked about them. Additionally, many used traditional healing practices to varying extents; some suggested that screening messages would be more acceptable if they acknowledged traditional practices. Participants did not detail how this might work, but this might be because focus groups may not have been an appropriate setting for such discussion. However, on the basis of other literature (7), acknowledging cultural factors might make screening messages more acceptable.

Participants also spent a lot of time discussing the health care system, particularly the IHS. Most of our participants used the IHS for at least some health care even if they had health insurance. Many IHS clinics rely on “contract health” to provide specialty services (including colonoscopy) at other facilities. This system provides access to advanced services not otherwise available, but the process can be complex. Having to navigate an additional system can compound the difficulties of screening.

Many men in our study were supportive of CRC screening and early detection. However, they said generic messages may not be effective, instead advocating for interventions that were “by the people, for the people.” That is, they asked for programs that were delivered by AIs, at culturally accessible venues, and addressed barriers found in native communities. Such culturally relevant efforts could ultimately reduce existing cancer disparities. We are working with our community partners to develop interventions in response to these concerns.

## Acknowledgments

This work was supported by the National Institutes of Health, National Center for Minority Health and Health Disparities (P20 MD004805, PIs: Daley, Greiner) and National Institutes of Health, National Cancer Institute (R03CA121828, PI: Daley). Dr James’s time was supported by Washington University School of Medicine, the Barnes-Jewish Hospital Foundation, and Siteman Cancer Center and the National Institutes of Health, National Cancer Institute (U54-CA153460, PI: Colditz).
